# Bacteremia caused by *Enterobacter asburiae* misidentified biochemically as *Cronobacter sakazakii* and accurately identified by matrix-assisted laser desorption/ionization time-of-flight mass spectrometry: a case report

**DOI:** 10.1186/s13256-021-03241-2

**Published:** 2022-01-19

**Authors:** Noboru Horinouchi, Seiji Shiota, Takeshi Takakura, Atsushi Yoshida, Ken Kikuchi, Akira Nishizono, Eishi Miyazaki

**Affiliations:** 1 Department of General Medicine, Almeida Memorial Hospital, 1509-2 Miyazaki, Oita, Oita 870-1195 Japan; 2 Department of General Medicine, Faculty of Medicine, Oita University, 1-1 Idaigaoka Hasama-machi, Yufu, Oita 879-5593 Japan; 3 Department of Infectious Diseases, Tokyo Women’s Medical University, 8-1 Kawada-cho, Shinjuku-ku, Tokyo, 162-8666 Japan; 4 Department of Microbiology, Faculty of Medicine, Oita University, 1-1 Idaigaoka, Hasama-machi, Yufu, Oita 879-5593 Japan

**Keywords:** *Enterobacter asburiae*, *Cronobacter sakazakii*, Matrix-assisted laser desorption/ionization time-of-flight mass spectrometry

## Abstract

**Background:**

Biochemical analyses of causative bacteria do not always result in clear identification, and new technologies aimed at improving diagnostic accuracy continue to be developed. Matrix-assisted laser desorption/ionization time-of-flight mass spectrometry is a rapid and accurate technique for bacterial identification. Misidentification of *Cronobacter sakazakii* is related to clinical and industrial problems. Here, we encountered a case of rare bacteremia in which the causative organism *Enterobacter asburiae* was biochemically misidentified as *C. sakazakii* before being correctly identified by matrix-assisted laser desorption/ionization time-of-flight mass spectrometry.

**Case presentation:**

An 87-year-old Asian man with no diabetes or active disease developed bacteremia and was admitted to our hospital. While the route of infection could not be determined despite various examinations, the clinical course was good following antibiotic therapy. Biochemical analyses identified the causative organism as *C. sakazakii*, but colonies on the blood agar medium showed a grayish coloration, differing from the yellowish coloration of typical *Cronobacter* colonies. Matrix-assisted laser desorption/ionization time-of-flight mass spectrometry was therefore performed, identifying the bacterium as *E. asburiae* on three independent analyses. This result was confirmed by multilocus sequence analysis using five housekeeping genes.

**Conclusions:**

Matrix-assisted laser desorption/ionization time-of-flight mass spectrometry may reduce misidentification of bacteria as *C. sakazakii* and improve the reporting rate of *E. asburiae*. This technique should be considered when biochemical bacterial misidentification is suspected.

## Background

Identification of the causative bacteria for infectious diseases using biochemical examinations is sometimes inaccurate, and new technologies aimed at improving diagnostic accuracy continue to be developed. Matrix-assisted laser desorption/ionization time-of-flight mass spectrometry (MALDI-TOF MS) allows rapid, accurate identification of the causative organism. This technique analyzes the patterns of proteins that are extracted from bacteria, which can reveal bacteria at the genus, species, and sometimes even subspecies levels [1].

Both *Cronobacter sakazakii* and *Enterobacter asburiae* are Gram-negative, rod-shaped, motile bacteria. *C. sakazakii* is an opportunistic pathogen that can cause lethal infection in newborns and the elderly, so accurate identification is crucial and misidentification by biochemical examination represents a critical problem [2]. *C. sakazakii* was initially identified under the genus *Enterobacter* before being recategorized under the genus *Cronobacter* [3]. Distinguishing between *Cronobacter* and *Enterobacter* is difficult due to the similarities in biochemical phenotypes [2]. Rapid, reliable identification of genus *Cronobacter* and differentiation from genus *Enterobacter* is important for epidemiological research. Here, we report a case of bacteremia in which the causative *E. asburiae* was initially misidentified as *C. sakazakii* by biochemical analyses before eventual correct identification by MALDI-TOF MS.

## Case presentation

**Fig. 1:**
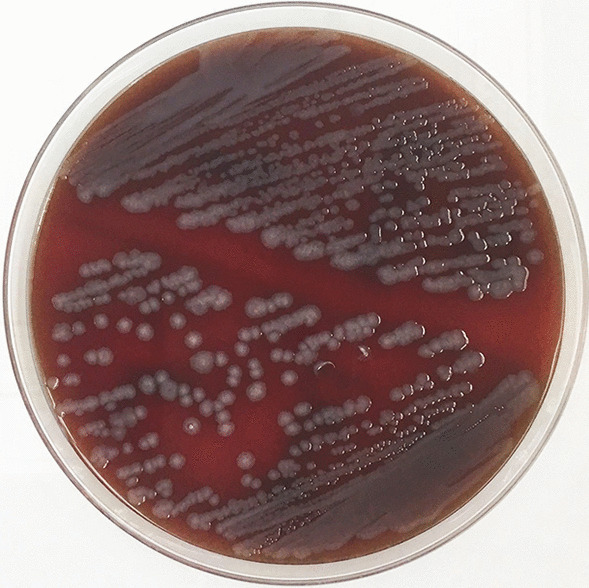
Colonies on blood agar medium. Colonies show grayish coloration rather than the characteristic yellow coloration of *C. sakazakii*

An 87-year-old Asian man visited his primary-care physician with a 3-day history of fever and severe malaise. He had no history of diabetes mellitus or active disease. He was admitted and treated with intravenous meropenem (MEPM) at 1 g/day and oral levofloxacin at 500 mg/day by his primary-care physician on day 1. The next day, a Gram-negative bacillus was detected from two sets of blood culture bottles. The patient was then transferred to our hospital for further examination and treatment. He was conscious. Body temperature was 37.7 °C, heart rate was 80 beats per minute, respiratory rate was 14 breaths per minute, and blood pressure was 113/70 mmHg. There were no significant findings on physical examination, chest X-ray, plain computed tomography of the head, whole-body contrast-enhanced computed tomography, transthoracic echocardiography, or colonoscopy. Blood testing revealed a white blood cell count of 8080 cells/μl, a C-reactive protein (CRP) level of 18.01 mg/dl, and a procalcitonin level of 1.02 ng/ml. Blood and urine cultures were negative. Intravenous MEPM (3 g/day) was administered at our hospital owing to a lack of improvement on the previous treatment (day 2). He was afebrile on day 3. CRP level decreased to 4.05 mg/dl on day 5. A blood culture taken by the previous physician reportedly revealed *C. sakazakii*. Antibiotic therapy was changed to ceftriaxone at 2 g/day based on the results of antimicrobial susceptibility testing on day 6 (Table 1). This treatment was stopped on day 10 after confirming normalization of CRP levels and negative results for blood culture. After discontinuation of antibiotic therapy and discharge from our hospital, the patient showed good progress without recurrence of fever. Biochemical analyses using the MicroScan WalkAway 96 system (Beckman Coulter, Brea, CA, USA) identified the causative organism as *C. sakazakii* (Table 2)*.* However, the colonies on the blood agar medium unexpectedly showed grayish coloration, differing from the yellowish coloration of typical *Cronobacter* colonies (Fig. 1). MALDI-TOF MS using Microflex LT with the Biotyper v3.1 database (Bruker Daltonics, Bremen, Germany) was therefore conducted. MALDI-TOF MS identified the bacterium as *E. asburiae* on three independent analyses (log score values of 2.19, 2.08, and 2.20, matching with *E. asburiae* type strain DSM 17506). Finally, multilocus sequence analysis using five housekeeping genes (*fusA*, *gyrB*, *leuS*, *rpoB*, and *hsp60*) [4] confirmed our isolate as *E. asburiae* (TWCC 57976). Nucleotide sequence data reported are available under the DNA Data Bank of Japan (DDBJ) accession numbers LC427844 to LC427849.

**Table 1:** Antibacterial susceptibility test results of the strain isolated in the present case

Antimicrobial agent	MIC (µg/ml)	Interpretation according to CLSI 2012 criteria
Ampicillin	> 16	R
Piperacillin	≤ 16	S
Cefazolin	> 16	R
Cefotiam	> 16	R
Cefotaxime	≤ 1	S
Cefepime	≤ 8	S
Imipenem/cilastatin	≤ 1	S
Gentamicin	≤ 4	S
Minocycline	≤ 4	S
Levofloxacin	≤ 2	S

MIC, minimum inhibitory concentration; CLSI, Clinical and Laboratory Standards Institute; R, resistant; S, susceptible.

**Table 2:** Biochemical phenotypes of *E. asburiae* and *C. sakazakii* in reference to the MicroScan WalkAway 96 system and results for the strain isolated in the present case

	*E. asburiae*	*C. sakazakii*	Present case
Glucose	99	99	+
Sucrose	99	99	+
Sorbitol	99	5	−
Raffinose	50	90	+
Rhamnose	5	99	+
Arabinose	95	99	+
Inositol	10	75	+
Adonitol	1	1	−
Melibiose	5	90	+
Urease	1	1	−
Hydrogen sulfide	1	1	−
Indole	1	1	−
Lysine decarboxylase	1	1	−
Arginine decarboxylase	25	75	−
Ornithine decarboxylase	95	95	+
Tryptophan deaminase	1	1	−
Esculin hydrolysis	90	95	+
Voges–Proskauer	1	95	+
Citrate	25	99	+
Malonate	1	10	−
β-Galactosidase	99	99	+

Each number represents the probability of biochemical reaction.

## Discussion and conclusions

In this case, biochemical analyses misidentified *E. asburiae* as *C. sakazakii*. As *C. sakazakii* can contaminate powdered infant formula and may cause fatal infections in newborns, biochemical screening for *Cronobacter* contamination is recommended [5]. Although accurate identification of *C. sakazakii* is extremely important to prevent potentially fatal infections of newborns, a previous study suggested that biochemical test panels are unreliable for identifying *Cronobacter* species [6]. To avoid misidentification, methods other than biochemical analyses need to be considered.

The biochemical misidentification in this case may have been caused by biochemical heterogeneity of bacteria. In the MicroScan WalkAway system, negativity for sorbitol fermentation and positivity for melibiose, rhamnose, and inositol fermentation and the Voges–Proskauer reaction matched the typical biochemical phenotype of *C. sakazakii*, but not that of *E. asburiae*. *E. asburiae* belongs to the *Enterobacter cloacae* complex. Six species of bacteria belong to this complex, which share similar biochemical phenotypes [7]. *C. sakazakii* was previously categorized as *Enterobacter sakazakii*, but was later recategorized into genus *Cronobacter* [3]. A previous report described *C. sakazakii* biochemically misidentified as *E. hormaechei*, belonging to the *E. cloacae* complex [8]. They suggested that misidentification of *E. hormaechei* as *C. sakazakii* may cause unnecessary financial losses for manufacturing companies. The activities of enzymes that determine the biochemical phenotype are heterogeneous and can cause misidentification of *Cronobacter* [9]. Biochemical misidentification among related strains has been reported in other bacteria, such as *Pseudomonas aeruginosa* [10] and enterohemorrhagic *Escherichia coli* [11]. Such misidentifications can lead to the prescription of inappropriate antibiotics and serious infection. Additional tests in consideration of the possibility of misidentification by biochemical analyses should be considered.

MALDI-TOF MS is a mass spectrometry technique that involves ionizing a sample using laser light and patterning constituent molecules of the target protein. Bacteria can be identified by comparing the constituent molecular pattern (mass spectrum) of the obtained material with a database [1]. This method is simple and quick compared with DNA-sequence-based methods. The effects of reducing hospitalization days and mortality rates through the proper use of antibiotics based on MALDI-TOF MS results have also been reported [12]. Further, MALDI-TOF MS allows quick identification of bacteria that show poor cultivation rates or long cultivation periods, improving the bacterial identification rate [13].

In our case, *E. asburiae* was initially biochemically misidentified as *C. sakazakii* before correct identification by MALDI-TOF MS. Although the significance of *E. asburiae* bacteremia has not been elucidated, its prevalence may be underestimated due to the misidentification of *E. asburiae* bacteremia. MALDI-TOF MS has the possibility of reducing misidentification and improving reporting rates of *E. asburiae.* This technique may also help elucidate the natural history of the bacteria. When biochemical misidentification of bacteria is suspected, MALDI-TOF MS should be considered for rapid and accurate identification.

## Data Availability

Not applicable.

## Abbreviations

MALDI-TOF MS: Matrix-assisted laser desorption/ionization time-of-flight mass spectrometry
MEPM: Meropenem
CRP: C-reactive protein

## References

[1] P Rahi O Prakash YS Shouche 2016 Matrix-assisted laser desorption/ionization time-of-flight mass-spectrometry (MALDI-TOF MS) based microbial identifications: challenges and scopes for microbial ecologists Front Microbiol 7 1359 10.3389/fmicb.2016.01359 PMC5003876 27625644

[2] R Stephan D Ziegler V Pfluger G Vogel A Lehner 2010 Rapid genus- and species-specific identification of *Cronobacter* spp. by matrix-assisted laser desorption ionization-time of flight mass spectrometry J Clin Microbiol 48 2846 2851 10.1128/JCM.00156-10 PMC2916578 20554814

[3] JJ Farmer 3rd 2015 My 40-year history with *Cronobacter*/*Enterobacter sakazakii*—lessons learned, myths debunked, and recommendations Front Pediatr 3 84 10.3389/fped.2015.00084 PMC4662064 26640778

[4] A Paauw MP Caspers FH Schuren MA Leverstein-van Hall A Deletoile RC Montijn 2008 Genomic diversity within the *Enterobacter cloacae* complex PLoS ONE 3 e3018 10.1371/journal.pone.0003018 PMC2515634 18716657

[5] Y Chen KY Song EW Brown KA Lampel 2010 Development of an improved protocol for the isolation and detection of *Enterobacter sakazakii* (*Cronobacter*) from powdered infant formula J Food Prot 73 1016 1022 10.4315/0362-028x-73.6.1016 20537255

[6] EE Jackson SJ Forsythe 2016 Comparative study of *Cronobacter* identification according to phenotyping methods BMC Microbiol 16 146 10.1186/s12866-016-0768-6 PMC4940867 27401027

[7] ML Mezzatesta F Gona S Stefani 2012 *Enterobacter cloacae* complex: clinical impact and emerging antibiotic resistance Future Microbiol 7 887 902 10.2217/fmb.12.61 22827309

[8] SM Townsend E Hurrell J Caubilla-Barron C Loc-Carrillo SJ Forsythe 2008 Characterization of an extended-spectrum beta-lactamase *Enterobacter hormaechei* nosocomial outbreak, and other *Enterobacter hormaechei* misidentified as *Cronobacter* (*Enterobacter*) *sakazakii* Microbiology. 154 3659 3667 10.1099/mic.0.2008/021980-0 19047733

[9] P Druggan C Iversen 2009 Culture media for the isolation of *Cronobacter* spp Int J Food Microbiol 136 169 178 10.1016/j.ijfoodmicro.2009.09.008 19896231

[10] N Wellinghausen J Kothe B Wirths A Sigge S Poppert 2005 Superiority of molecular techniques for identification of Gram-negative, oxidase-positive rods, including morphologically nontypical *Pseudomonas aeruginosa*, from patients with cystic fibrosis J Clin Microbiol 43 4070 4075 10.1128/JCM.43.8.4070-4075.2005 PMC1233906 16081953

[11] LK Crawford-Miksza S Himathongkham ML Dodd AS Badoiu OM Badoiu LS Guthertz 2009 Misidentification of a variant biotype of *Escherichia coli* O157:H7 as *Escherichia fergusonii* by Vitek 2 Compact J Clin Microbiol 47 872 873 10.1128/JCM.02425-08 PMC2650936 19116349

[12] AM Huang D Newton A Kunapuli TN Gandhi LL Washer J Isip 2013 Impact of rapid organism identification via matrix-assisted laser desorption/ionization time-of-flight combined with antimicrobial stewardship team intervention in adult patients with bacteremia and candidemia Clin Infect Dis 57 1237 1245 10.1093/cid/cit498 23899684

[13] S Biswas JM Rolain 2013 Use of MALDI-TOF mass spectrometry for identification of bacteria that are difficult to culture J Microbiol Methods 92 14 24 10.1016/j.mimet.2012.10.014 23154044

